# Supplementary material for: As the Pandemic Progresses, How Does Willingness to Vaccinate against COVID-19 Evolve?
Source: Int J Environ Res Public Health. 2021 Jan 19;18(2):797. doi: 10.3390/ijerph18020797 (PMC7832839; doi:10.3390/ijerph18020797)
Supplement: Supplementary file 1 [file ijerph-18-00797-s001.zip › final/Supplementary file 1.docx]

**Supplementary file 1. Survey questions used to assess demographics, media use and willingness to vaccinate.**

1. In what year where you born?

_______________

1. What is your gender?
   1. Male
   2. Female
   3. Non-binary
   4. Prefer not to say
2. What is the postcode of your residential address?

_______________

1. What is the highest level of education you have completed?
   1. No schooling
   2. Year 10 or below
   3. Year 11 or 12
   4. Technical studies / Trade certificate / Diploma / Advanced Diploma
   5. Bachelor Degree / Graduate Diploma
   6. Postgraduate including Masters or PhD
2. What is the total of all wages/salaries, government benefits, pensions, allowances and other income **received by all persons in your household (including yourself)**, per week?
   1. $3,000 or more per week ($156,000 or more per year)
   2. $2,000 - $2,999 per week ($104,000 - $155,999 per year)
   3. $1,750 - $1,999 per week ($91,000 - $103,999 per year)
   4. $1,500 - $1,749 per week ($78,000 - $90,999 per year)
   5. $1,250 - $1,499 per week ($65,000 - $77,999 per year)
   6. $1,000 - $1,249 per week ($52,000 - $64,999 per year)
   7. $800 - $999 per week ($41,600 - $51,999 per year)
   8. $650 - $799 per week ($33,800 - $41,599 per year)
   9. $500 - $649 per week ($26,000 - $33,799 per year)
   10. $400 - $499 per week ($20,800 - $25,999 per year)
   11. $300 - $399 per week ($15,600 - $20,799 per year)
   12. $150 - $299 per week ($7,800 - $15,599 per year)
   13. $1 - $149 per week ($1 - $7,799 per year)
   14. Nil income
   15. Prefer not to say
3. Have you ever been told by a doctor that you have any chronic health problems?
   1. Yes
   2. No
4. How much do you **currently** use social media (e.g. Facebook, Reddit, Twitter)?
   1. Never
   2. Rarely
   3. Less than 1 hour a day
   4. 1-2 hours a day
   5. 3-4 hours a day
   6. 5-6 hours a day
   7. 7-8 hours a day
   8. Most of the day
5. How much do you **currently** listen to/read/watch news on the radio, newspaper or TV?
   1. Never
   2. Rarely
   3. Less than 1 hour a day
   4. 1-2 hours a day
   5. 3-4 hours a day
   6. 5-6 hours a day
   7. 7-8 hours a day
   8. Most of the day
6. Please indicate your level of agreement with the following statements

|  | Strongly agree (5) | Agree (4) | Neither agree nor disagree (3) | Disagree (2) | Strongly disagree (1) |
| --- | --- | --- | --- | --- | --- |
| If a new vaccine for COVID-19 was released **that was proven to be safe and effective**, I would get vaccinated immediately |  |  |  |  |  |
| If a new vaccine for COVID-19 was released **but had not yet been proven to be safe and effective**, I would get vaccinated immediately |  |  |  |  |  |
| Even before the COVID-19 pandemic, I have always vaccinated myself against diseases when recommended by health professionals |  |  |  |  |  |
