# Supplementary material for: As the Pandemic Progresses, How Does Willingness to Vaccinate against COVID-19 Evolve?
Source: Int J Environ Res Public Health. 2021 Jan 19;18(2):797. doi: 10.3390/ijerph18020797 (PMC7832839; doi:10.3390/ijerph18020797)
Supplement: Supplementary file 1 [file ijerph-18-00797-s001.zip › final/Supplementary file 2.docx]

**Supplementary file 2. Usual willingness to receive vaccinations recommended by doctor by demographics, chronic disease status and media use**

**Table S1.** Multinomial logistic regression of usual willingness to receive vaccinations recommended by doctor by time, demographics, chronic disease status and media use (*n* = 2328).

| **Demographics and Time** | **Variable Categories** | **Agree**  **OR (95% CI)** | **Neither Agree nor Disagree**  **OR (95% CI)** |
| --- | --- | --- | --- |
| Time | Survey 2 | 1.11 (0.77–1.60) | 1.04 (0.64–1.72) |
|  | Survey 1 | 1.00 | 1.00 |
| Living Location | Victoria | 0.97 (0.61–1.55) | 0.69 (0.35–1.37) |
|  | Other Australian state or territory | 1.00 | 1.00 |
| Time · Location | Victoria | 0.73 (0.38–1.42) | 0.70 (0.25–1.91) |
|  | Other Australian state or territory | 1.00 | 1.00 |
| Age (years) | 18–34 | 1.81 (1.04–3.14) * | 2.61 (1.22–5.58) * |
|  | 35–44 | 1.09 (0.66–1.78) | 1.50 (0.72–3.13) |
|  | 45–54 | 0.94 (0.60–1.49) | 1.75 (0.91–3.40) |
|  | 55–64 | 0.69 (0.46–1.02) | 1.28 (0.70–2.32) |
|  | ≥65 | 1.00 | 1.00 |
| Gender | Female | 1.17 (0.87–1.56) | 0.96 (0.64–1.44) |
|  | Male | 1.00 | 1.00 |
| Education | Year 12 or below | 0.62 (0.41–0.92) * | 1.37 (0.81–2.34) |
|  | Technical studies, Certificate, Diploma | 0.51 (0.37–0.71) | 0.99 (0.63–1.57) |
|  | Bachelor degree or above | 1.00 | 1.00 |
| Chronic disease status | With chronic disease | 1.88 (1.40–2.53) *** | 1.31 (0.87–1.97) |
|  | Without chronic disease | 1.00 | 1.00 |
| Social media use | <1 h | 1.23 (0.83–1.84) | 0.95 (0.54–1.65) |
|  | 1–2 h | 1.03 (0.75–1.42) | 0.73 (0.46–1.15) |
|  | >3 h | 1.00 | 1.00 |
| Traditional media use | <1 h | 0.52 (0.35–0.78)** | 0.68 (0.40–1.17) |
|  | 1–2 h | 0.92 (0.62–1.36) | 0.81 (0.47–1.40) |
|  | >3 h | 1.00 | 1.00 |

Reference category = Disagree. *** *p* < 0.001, ** *p* < 0.01, * *p* < 0.05.
